# ThongPaDisp: An open-source 3D-printed shuttlecock dispenser using a Grip-Gate-Push mechanism

**DOI:** 10.1016/j.ohx.2026.e00783

**Published:** 2026-04-29

**Authors:** Thanawat Saeeab, Wichuda Phiphitphibunsuk

**Affiliations:** a School of Information and Communication Technology, University of Phayao, Phayao 56000, Thailand; b School of Pharmaceutical Sciences, University of Phayao, Phayao 56000, Thailand

**Keywords:** Badminton training, Shuttlecock dispenser, Jamming mitigation, Feather deformation, 3D printing

## Abstract

This paper presents ThongPaDisp, a low-cost, open-source, 3D-printed shuttlecock dispenser developed to automate shuttlecock feeding. The Grip-Gate-Push mechanism implemented in ThongPaDisp overcomes the limitations of conventional Grip-Gate (gravity-only) dispensing systems, which are prone to jamming caused by feather deformation and interlocking within the shuttlecock stack. The device is a modular feeder designed for integration with a shuttlecock launcher or standalone operation. The extraction sequence is actuated by three independent servo motors synchronized via a microcontroller. Two operating modes are supported: (1) automatic dispensing triggered by hand detection beneath the device, and (2) manual control via a wireless infrared remote for testing. The experimental evaluation utilized both new and used shuttlecocks across five repeated-dispensing trials. Under the Grip-Gate (gravity-only) configuration, jamming occurred in 3.3% (95% CI: [0.0%, 11.9%]) of trials for new shuttlecocks and 40.0% (95% CI: [11.9%, 68.2%]) of used shuttlecocks, with a mean fall time of 0.29 s. Conversely, the full Grip-Gate-Push configuration significantly improved reliability, exhibiting 0% and 1.7% (95% CI: [0.0%, 5.4%]) jamming for new and used shuttlecocks, respectively, with a mean release time of 0.61 s. The lightweight ThongPaDisp design (0.45 kg) is released as open-source hardware to enable easy fabrication, modification, and further development.

## Highlights

• Reliable shuttlecock dispensing via novel Grip-Gate-Push (GGP) mechanism.
• Dual-mode activation: automatic hand-triggering and wireless remote control.
• Consistent release time (≈0.61 s) for both new and used shuttlecocks.
• Lightweight (0.45 kg), low-cost (≈49.50 USD), and fully open-source hardware.

## Graphical abstract

**Figure:**
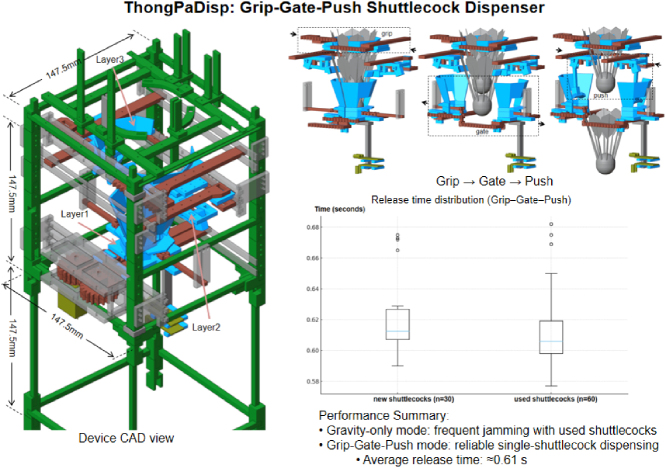

## Specifications table

**Table:** 

Hardware name	ThongPaDisp

Subject area	• Engineering and material science • Educational tools and open source alternatives to existing infrastructure

Hardware type	• Other (sports training dispenser; automated feeder for shuttlecocks)

Closest commercial analog	Examples include commercial shuttlecock holders such as the S.Holder [1] and the BadmintonHQ 50 cm shuttlecock tube [2].

Open source license	CERN-OHL-S v2 (hardware design files); CC BY 4.0 (documentation); MIT (firmware/software)

Cost of hardware	Approx. 49.50 USD.

Source file repository	https://doi.org/10.5281/zenodo.18899461

	

## 1 Hardware in context

Badminton launching machines are widely used in athletic training to reduce coaching workload and improve the consistency of shuttlecock delivery during repetitive drills. In conventional training routines, coaches typically deliver shuttlecocks manually by stacking multiple shuttlecocks in one hand and releasing them sequentially with the other, while adjusting the delivery rhythm to match the athlete’s skill level. However, manual routines are often constrained by limited variability and predictable patterns [3], while also requiring strict temporal control to manage athlete workload [4]. Furthermore, manual feeding can lead to coach muscle fatigue, inconsistent delivery, and a long-term decline in training effectiveness. Although assistive devices such as floor-standing vertical tubes [1] or shoulder-mounted dispensers [2] are available, they still rely heavily on manual labor. Consequently, the advent of automatic launchers [5] has marked a significant shift in training methodologies, necessitating the development of automated dispensing technologies that improve consistency and reduce physical strain.

Recent advancements in low-cost dispensing systems, especially those using 3D printing, have made it easier to create automated devices that are accessible, customizable, and repeatable for training and laboratory use. Several studies have examined novel approaches to improve dispensing systems. One study [6] developed a low-cost, open-source 3D-printed antibody dispenser for lateral flow assay strips, demonstrating that 3D printing can produce reliable dispensing systems. Furthermore, recent advancements in affordable 3D printing for laboratory automation emphasize that open-source printed components can deliver precise and stable mechanical performance [7]. Other dispensing systems rely primarily on microcontroller-based control for precise mechanical sequencing. In liquid handling, the SALAD system [8] presents a syringe-based, Arduino-operated dispenser that provides precise flow control. Additional research [9] implemented an Arduino-based liquid-delivery controller capable of pushing and pulling defined volumes with high precision, while other work [10] focused on an atomic force microscope with an Arduino controller to coordinate its mechanical movements with accurate timing.

Dispensing shuttlecocks presents distinct mechanical challenges due to their conical geometry and the tendency of feathers to interlock when stacked. Commercial training machines, such as the Siboasi SS-B7 [11], employ a singulation mechanism that can be conceptually described as a “Grip-Prepare-Release” sequence. In this architecture, paired semi-circular gripping elements synchronously adjust their aperture to secure the shuttlecock cork (“grip”), expand beyond the shuttlecock diameter to allow controlled transitional alignment (“prepare”), and subsequently release it to allow gravity-driven descent toward the propulsion stage (“release”). In addition, the hopper typically incorporates a tapered neck geometry that stabilizes the feather skirts and maintains vertical alignment during stacking.

In the patent literature, one system [12] uses a weighted pendulum to apply pressure on a stack of shuttlecocks. This pendulum adjusts its position as the stack height changes, pushing the lowest shuttlecock out. Another commonly reported method uses two counter-rotating shafts that grip the shuttlecock from both sides and eject it with synchronized movement. Certain Thai patents [13], [14] describe a 3D-printed sorting mechanism that separates shuttlecocks using a three-layer structure with minimal error. Previous experimental work [15] demonstrated that this sorting section consistently released new and used shuttlecocks with high reliability. This indicates that the mechanism effectively separates the lowest shuttlecock, even when evaluated independently from the launching module.

Despite these developments, no open-source platform currently exists that integrates this sorting mechanism into a fully functional device capable of reliably dispensing both new and used shuttlecocks. This work introduces *ThongPaDisp*, a modular 3D-printed device built from independently assembled components. The system employs a unified Grip-Gate-Push (GGP) sequence controlled by three servos synchronized via an Arduino microcontroller to release one shuttlecock at a time from a vertical stack with minimal user interaction. The system can be activated either by detecting a user’s hand at the release point or via an infrared remote control. This study compares a passive Grip-Gate (gravity-only) configuration with a full Grip-Gate-Push mode to evaluate the role of push-assisted separation. Rather than proposing a fundamentally new mechanical separation principle, the contribution of this work lies in the complete open-source integration, modular redesign, and reproducible implementation of the Grip-Gate-Push architecture for shuttlecock dispensing. All design files, firmware, and assembly documentation are openly released to support replication, modification, and further development.

## 2 Hardware description

### 2.1 Overview

**Fig. 1:**
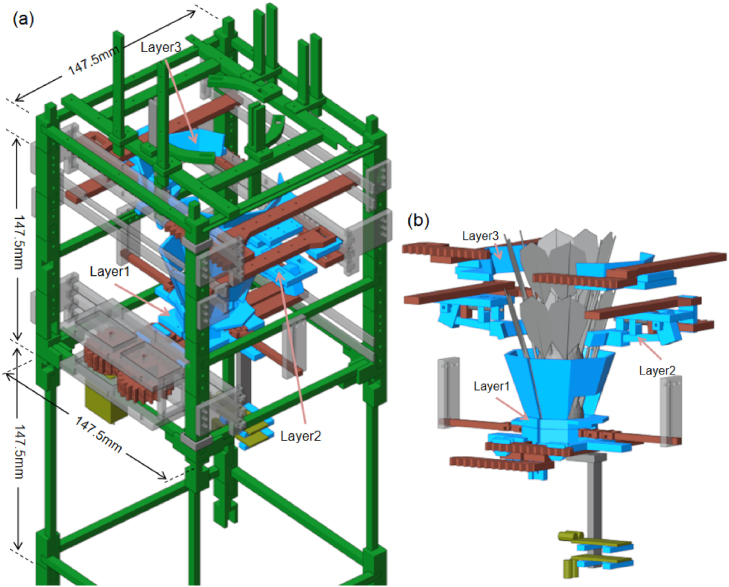
Overview of the ThongPaDisp system: (a) Fully assembled device showing the three-layer Grip-Gate-Push (GGP) extraction structure integrated with the supporting frame, and (b) Exploded view illustrating the internal arrangement of Layer 1 (Gate), Layer 2 (Pusher), and Layer 3 (Gripper).

ThongPaDisp is designed to load shuttlecocks from the top and release them individually through a vertical column using a three-stage Grip-Gate-Push (GGP) mechanism. Fig. 1(a) illustrates the complete assembled system, while Fig. 1(b) illustrates the internal arrangement of the three functional layers. Each layer is actuated by an independent servo-driven arm, allowing sequential motion without mechanical interference between stages. The actuation arms share a common base geometry, while layer-specific attachments define their respective functions. A microcontroller coordinates the timing of each stage, ensuring safe and repeatable operation. The system supports two interaction modes: automatic dispensing triggered by a hand-detection sensor and manual operation via an infrared (IR) remote control. In practical use, the device is placed on a tabletop, allowing users to retrieve each released shuttlecock consistently during training.

### 2.2 Frame and structural modules

**Fig. 2:**
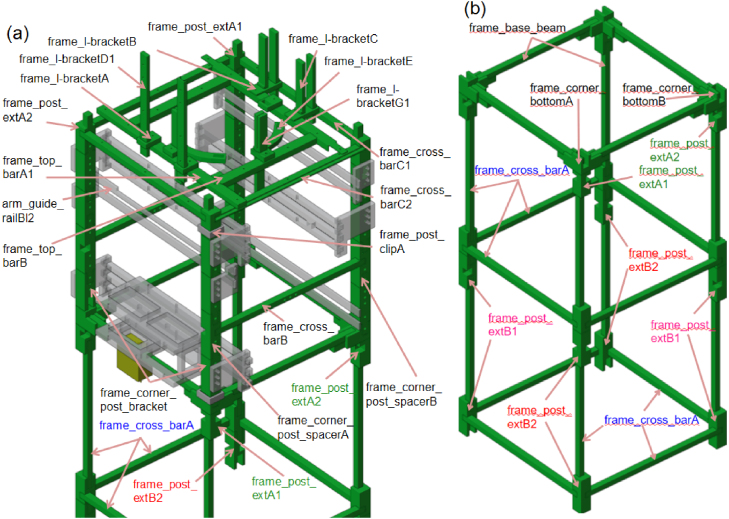
Frame and structural modules of the ThongPaDisp: (a) Main frame structure highlighting vertical posts, crossbars, corner brackets, and accessory mounting points, and (b) Complete lower frame assembly showing base beams, crossbars, corner joints, and extension posts used to adjust the base height and provide vertical clearance beneath the dispensing outlet. Different colors are used only to improve visual separation between labeled components and to indicate corresponding parts across subfigures.

The primary support structure consists of a modular frame designed for sectional 3D printing and straightforward assembly, as shown in Fig. 2(a). The frame is composed of vertical posts connected by horizontal crossbars to form a rigid, rectangular structure. Each post is constructed from stacked extension segments, allowing the overall height of the device to be adjusted according to the number of shuttlecocks loaded or user preference. Integrated alignment features along the posts ensure accurate positioning of the arm drive modules and extraction layers. The upper section of the frame houses the Arduino controller, battery pack, and a shuttlecock container fabricated from a standard 1.5 L PET bottle. The lower section provides sufficient vertical clearance to enable operation on a standard tabletop while allowing hand placement beneath the dispensing outlet for automatic triggering, as shown in Fig. 2(b). Using modular extension posts, the base height of the frame can be adjusted to accommodate different operating environments or integration with underlying launching mechanisms. This open architecture also reduces the likelihood of rebound-related blockages and allows released shuttlecocks to exit the mechanism without obstruction.

### 2.3 Arm guiding and drive module

**Fig. 3:**
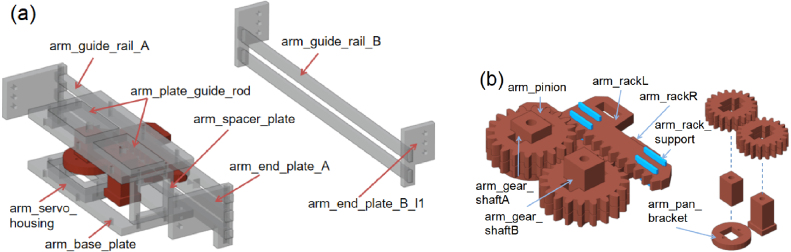
Arm guiding and drive mechanisms: (a) Arm guiding structure showing the base plate, guide rails, end plates, and guide rod, and (b) Rack-and-pinion transmission assembly comprising pinion gears, rack segments, and supporting shafts for linear actuation.

Each GGP stage is actuated by a dedicated arm module that converts servo rotation into controlled linear motion. As shown in Fig. 3(a), the arm is guided by proximal and distal linear rails that maintain alignment throughout the actuation stroke. Motion is generated by a compact SG90 servo motor driving a rack-and-pinion transmission, shown in Fig. 3(b). The servo’s rotational motion is transmitted through a pinion gear to dual rack segments, producing synchronized linear motion of the arm components. This configuration provides smooth motion transfer and stable contact forces during gripping, gating, and pushing operations. Servo motion ranges and motion sequences are defined in firmware, preventing mechanical collisions and ensuring repeatable actuation. The modular design enables the reuse of identical base components across all three stages.

### 2.4 Three-layer dispensing mechanism: Grip, Gate, and Push

**Fig. 4:**
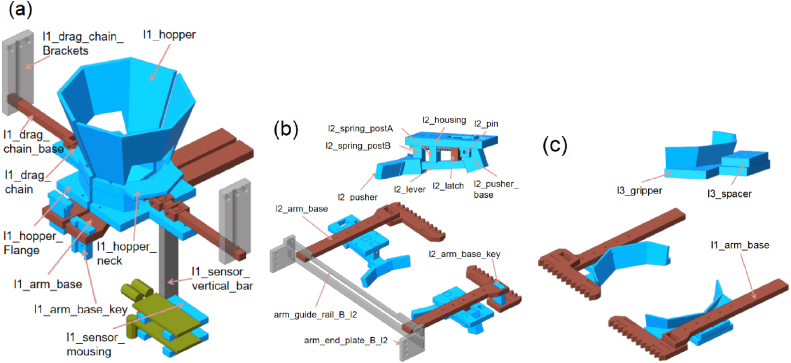
Three-layer extraction components: (a) Layer 1 (Gate) showing the hopper, neck, alignment chain, and sensor mounts, (b) Layer 2 (Pusher) showing the lever, latch, pusher, and spring-return mechanism, and (c) Layer 3 (Gripper) showing the feather-matched gripper geometry.

**Fig. 5:**
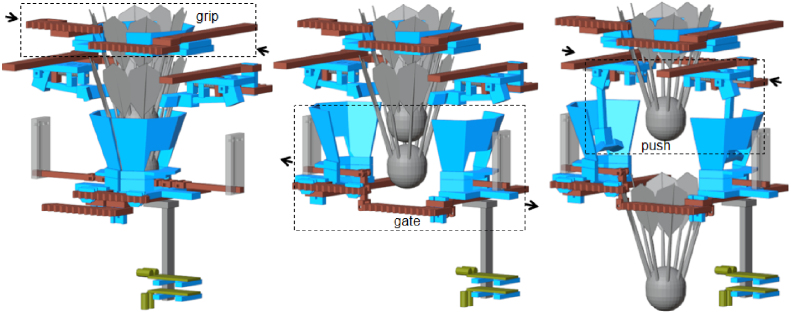
Grip-Gate-Push (GGP) dispensing sequence: the gripper (left) stabilizes the shuttlecock stack, the gate (center) opens to release the lowest shuttlecock, and the pusher (right) ejects it from the column.

The shuttlecock dispensing process is based on a three-stage Grip-Gate-Push mechanism, in which each stage performs a distinct and sequential functional role. Layer 1 (Gate) supports a column of shuttlecocks and actuates to open and close, allowing only the lowest shuttlecock to be released downward (Fig. 4(a)). An alignment chain is integrated, allowing the shuttlecock cork to maintain vertical orientation during release. Two infrared sensors are employed: one to detect the ejected shuttlecock as a passing object, and the other to sense the user’s hand for system activation. Layer 2 (Pusher) employs a single lever mechanism actuated by a spring, which ejects the released shuttlecock from the column and enables automatic return to its initial position (Fig. 4(b)). The forward motion of the pusher ejects the shuttlecock outward, whereas the reverse motion returns the mechanism to its initial position for the subsequent operating cycle. Layer 3 (Gripper) is used to stabilize the shuttlecock stack by gently holding the second-lowest shuttlecock during the dispensing process (Fig. 4(c)). The gripper geometry is designed to conform to the curvature of the shuttlecock feathers, providing sufficient restraint while preventing structural damage. In summary, the dispensing sequence consists of three steps: the gripper stabilizes the shuttlecock stack, the gate opens to release the lowest shuttlecock, and the pusher ejects the shuttlecock from the column, as shown in Fig. 5.

### 2.5 Control logic and timing

An Arduino Uno controls dispensing operations via infrared (IR) input and obstruction sensing. The IR remote commands are defined as follows:

• **Key 1:** Initiates continuous dispensing of three shuttlecocks.
• **Key 2:** Dispenses a single shuttlecock.
• **Key 4:** Executes the forward half-cycle (Grip-Gate-Push forward).
• **Key 5:** Executes the return half-cycle (Grip-Gate-Push return).

**Fig. 6:**
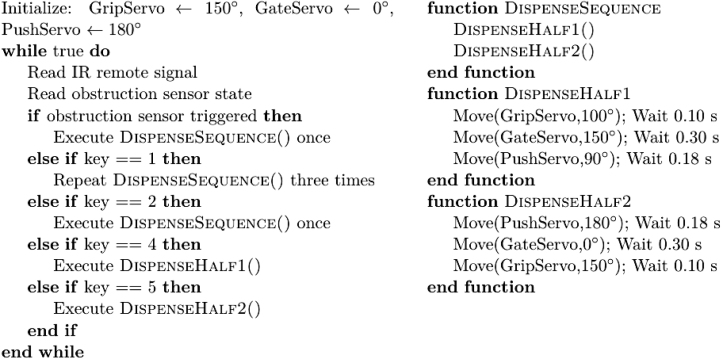
Algorithm for IR and sensor multi-mode control of the GGP dispensing mechanism.

Servo motion is calibrated such that a 60° movement takes approximately 0.12 s, preventing layer overlap. Typical wait times are 0.10 s for 50°, 0.18 s for 90°, and 0.30 s for 150°. To coordinate these timing constraints, the control logic is modularized as shown in Fig. 6.^1^ The main loop interprets IR commands for both full and half-cycle operations, while the obstruction sensor independently triggers a full dispensing cycle. The DispenseSequence() function performs a complete GGP cycle by sequentially calling DispenseHalf1() and DispenseHalf2(), allowing each half-cycle to be triggered independently for calibration or debugging purposes.

## 3 Design files summary

**Design files are openly available at DOI:** 10.5281/zenodo.18899461

To facilitate reproducibility of the ThongPaDisp system, Table 1 summarizes the primary functional modules of the Grip-Gate-Push (GGP) architecture. The complete inventory of all 3D-printable components, including structural elements and auxiliary brackets, has been relocated to the Supplementary Material (Tables S1–S5) to maintain clarity.

**Table 1:** Main modules and corresponding supplementary tables.

Module	Core functionality	Supplementary reference
Frame Structure	Provides structural support, alignment, and positional stability for the layered mechanism	Table S1 Frame structure design files
Arm Drive and Motion Transmission	Converts servo rotation into controlled linear displacement for actuation of the GGP sequence	Table S2 Arm drive and common motion component design files
Layer 1 (Gating and Sensing)	Supports the shuttlecock column and selectively releases the lowest shuttlecock while maintaining alignment and enabling infrared-based state detection	Table S3 Layer 1 (gating and sensing) design files
Layer 2 (Pushing and Separation)	Separates and ejects the released shuttlecock using a spring-assisted lever mechanism with automatic return	Table S4 Layer 2 (pushing and separation) design files
Layer 3 (Gripping)	Temporarily stabilizes the shuttlecock stack to prevent unintended multi-feed during gate actuation	Table S5 Layer 3 (gripping) design files

## 4 Bill of materials (BOM)

Table 2 lists the components along with their average prices in USD.

**Table 2:** Bill of materials (BOM).

Designator	Component	Number	Cost/Unit (USD)	Total Cost (USD)	Source of materials	Material type
Microcontroller Unit	Arduino Uno (R3-compatible)	1	6.00	6.00	Aliexpress	Semiconductor

Servo Motors	Micro Servo SG90 (9g)	3	1.50	4.50	Aliexpress	Composite

Battery Pack	3.7V 2000mAh Li-Po Battery	1	6.00	6.00	Aliexpress	Composite

Charging Module	Charging Module (Step-up)	1	1.50	1.50	Aliexpress	Semiconductor

IR Receiver Sensor	IR receiver module (38kHz)	1	1.00	1.00	Aliexpress	Semiconductor

Obstruction Sensors	Obstruction IR sensors	2	1.00	2.00	Aliexpress	Semiconductor

Adhesive	UHU Multi-purpose glue	1	3.50	3.50	Aliexpress	Polymer

Mechanical Springs	Springs 3mm x 20mm (pack)	1	2.00	2.00	Aliexpress	Metal

Fasteners (Screws)	Screws M2 x 12mm (pack)	1	2.00	2.00	Aliexpress	Metal

Structure (Bamboo)	Bamboo sticks 5x5x30mm (pack)	1	3.00	3.00	Aliexpress	Biomaterial

Structure (Wood)	Popsicle sticks 190x6x1.5mm (pack)	1	3.00	3.00	Aliexpress	Biomaterial

3D Printing Material	PLA+ Filament (0.5 kg est.)	0.5	30.00	15.00	Aliexpress	Polymer

**Total Cost**				**49.50**		

## 5 Build instructions

**Printed parts:** All 3D-printed components listed in this section are available in the Zenodo repository ( 10.5281/zenodo.18899461). While PLA+ provides a suitable baseline material for initial prototyping due to its stiffness, cost-effectiveness, and ease of printing, alternative thermoplastics such as PETG or ABS may be preferable for final builds intended for long-term, high-cycle, or elevated-temperature deployment. Typical print settings include a 0.40 mm nozzle with a 0.40 mm layer height and 20%–30% infill, which provide adequate strength for all structural and moving components regardless of the chosen filament.

In addition to the printed components, standard wooden toothpicks and bamboo skewers are used as functional mechanical elements. A 2 mm wooden toothpick is employed as an internal rotational shaft, as shown in Fig. 11(a), and also serves as a substitute for a long screw in the assembly illustrated in Fig. 10(b). Similarly, a 3 mm bamboo skewer functions as an internal rotational shaft for the joint illustrated in Fig. 9(a), providing sufficient stiffness while allowing smooth rotational motion.

**Fig. 7:**
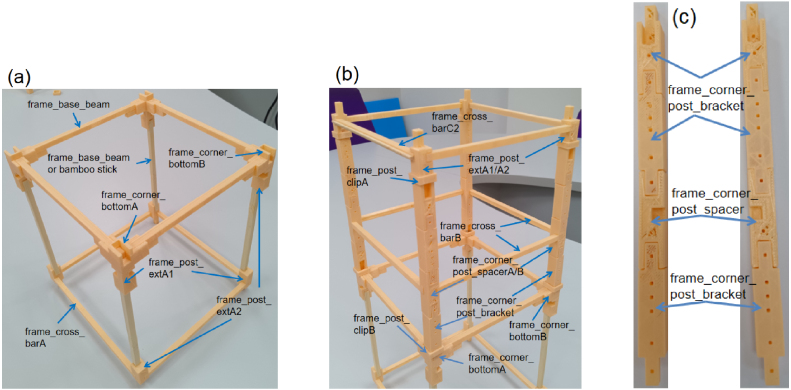
Frame assembly: (a) Base frame structure, (b) Three-level frame structure, and (c) Stacking sequence of the corner post bracket and spacer components.

**Fig. 8:**
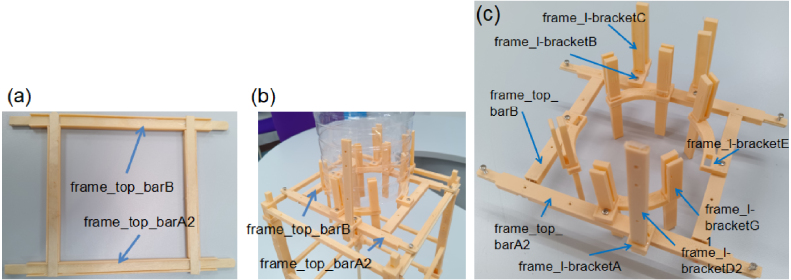
Top frame assembly: (a) Assembly of the top bars, (b) Installed top frame with a water bottle, and (c) Completed top frame.

**Reinforcement and structural considerations:** For parts l1_arm_baseA, l1_arm_base_keyA, and frame_cross_barB, additional reinforcement is recommended. This can be achieved by increasing the number of top and bottom layers and using at least two perimeter walls. Alternatively, reinforced STL versions are provided (e.g., l1_arm_baseB, l1_arm_base_keyB (Fig. 10), frame_l-bracketD2 (Fig. 8(c)), frame_cross_barC2 (Fig. 7(b)), frame_top_barA2 (Fig. 7(a)) and frame_top_barB (Fig. 7(a))), which feature internal channels designed to accommodate popsicle sticks. The sticks are inserted into the channels and bonded using adhesive to significantly increase structural rigidity. Additionally, the frame_cross_barA component can be substituted with 5 × 5 mm bamboo sticks (listed in the BOM), cut to 13.75 cm, as shown in Fig. 7(a).

**Assembly and bonding:** Connections between printed parts are formed using a multi-purpose adhesive. Adhesive bonding is suitable for fixed joints, while screws are recommended where alignment, disassembly, or maintenance access is required.

**Screw hole preparation:** For parts featuring pilot holes for screws, especially those reinforced with inserted sticks (e.g., popsicle sticks or bamboo sticks) that may partially block the screw channels, the holes should be reopened by drilling to the final diameter using a 2 mm drill bit and a hand drill prior to assembly.

**Table 3:** 3D-printed STL files and quantities used for the frame assembly.

STL File	Qty	STL File	Qty	STL File	Qty
frame_corner_bottomA	2	frame_corner_bottomB	2	frame_base_beam	4
frame_post_extA1	6	frame_post_extA2	6	frame_cross_barA	4
frame_cross_barB	4	frame_cross_barC1	2	frame_cross_barC2	2
frame_corner_post_bracket	12	frame_corner_post_spacerA	2	frame_corner_post_spacerB	2
frame_top_barA2	2	frame_top_barB	2	frame_l-bracketA	1
frame_l-bracketB	2	frame_l-bracketC	4	frame_l-bracketD2	1
frame_l-bracketE	4	frame_l-bracketG1	8	–	–

### 5.1 Frame assembly

• Fabricate all 3D-printed parts listed in Table 3.
• **Base Assembly:** Assemble the rectangular base using frame_corner_bottom, four frame_base_beams, frame_post_extA, and frame_cross_barA. Connect the lower and upper frame_post_extA components (in opposite orientation) using frame_base_beam or bamboo sticks as reinforcement. Attach each frame_post_extA to the frame_corner_bottom parts to form the corner joints (Fig. 7(a)).
• **Post Stacking:** Stack the components in the following order: one frame_corner_post_bracket, followed by one frame_corner_post_spacer, and then two additional frame_corner_post_bracket pieces. Apply adhesive between each layer to ensure firm bonding and vertical alignment (Fig. 7(c)).
• **Main Frame Integration:** Connect the four frame_post_extA columns on all sides using frame_cross_barC at the upper level and frame_cross_barB with frame_corner_post_spacer at the middle level. Place the assembled frame onto the base frame structure (Fig. 7(b)).
• **Top Frame:** Join frame_top_barA and frame_top_barB (Fig. 8(a)). Connect the top bars using frame_l-bracketB and frame_l-bracketC arranged perpendicularly. Mount this structure onto frame_top_barA. Install frame_l-bracketE and frame_l-bracketG (fixed perpendicularly). Finally, assemble frame_l-bracketA and frame_l-bracketD (Fig. 8(c)).
• Install the completed top frame. A 1.5 L water bottle serves as the shuttlecock container (Fig. 8(b)).

**Table 4:** 3D-printed STL files and quantities used for one arm module.

STL File	Qty	STL File	Qty	STL File	Qty
arm_base_plate	2	arm_guide_rail_A	1	arm_spacer_plate	2
arm_plate_guide_rod	3	arm_servo_housing	2	arm_pan_bracket	1
arm_gear_shaftA	1	arm_gear_shaftB	1	arm_rackL	1
arm_rackR	1	arm_pinion	4	arm_rack_support	8
arm_end_plate_A	2	–	–	–	–

### 5.2 Arm module

**Fig. 9:**
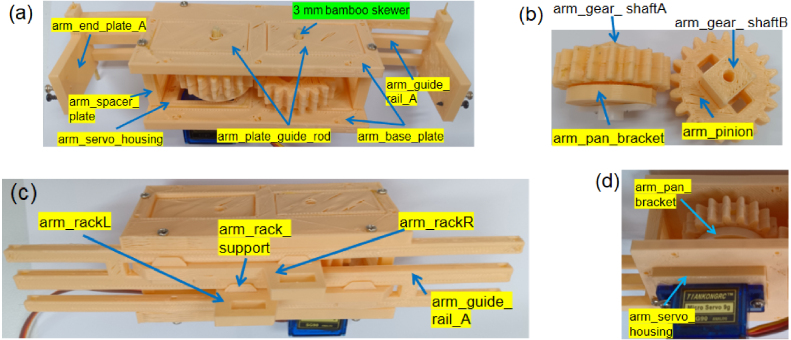
Arm module assembly: (a) Assembly of the arm base plates, guide rails, and spacer plates, (b) Pinion gear, (c) Installation of the rack-and-rail unit with support for alignment, and (d) Installation of the servo motor onto the drivetrain assembly.

• Fabricate all parts listed in Table 4.
• **Guide Assembly:** Attach arm_base_plate to both sides of arm_guide_railA. Install arm_spacer_plate pieces to form a stable rectangular frame (Fig. 9(a)).
• **Drivetrain:** Mount arm_pan_bracket on arm_gear_shaftA with two pinions. Attach two pinions to arm_gear_shaftB (Fig. 9(b)). Insert arm_rackL and arm_rackR (supported by arm_rack_support) into the guide rail. Close both sides with arm_end_plate_A (Fig. 9(c)).
• **Servo Installation:** Install an SG90 servo into arm_servo_housing. Mesh its output gear with the arm_pan_bracket gear to transmit rotational motion to the rack-and-pinion system (Fig. 9(d)).

**Table 5:** 3D-printed STL files and quantities used for the Layer 1 module.

STL File	Qty	STL File	Qty	STL File	Qty
l1_hopper	2	l1_hopper_neck	2	l1_hopper_flange	2
arm_guide_rail_B	2	arm_end_plate_B_l1	2	l1_arm_baseA	2
l1_arm_base_keyB	2	l1_sensor_vertical_bar	1	l1_sensor_housing	2
l1_drag_chain_bracket	2	l1_drag_chain	4	l1_drag_chain_base	2

### 5.3 Layer 1: Gating/sensing

**Fig. 10:**
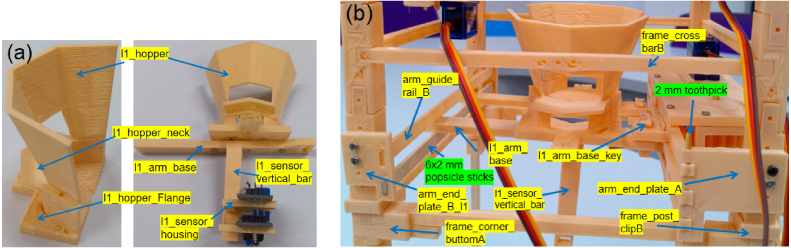
Layer 1 assembly: (a) Hopper construction, and (b) Integration onto the frame, with the arm module flipped so that the servo motor is positioned on the upper side for proper alignment.

**Fig. 11:**
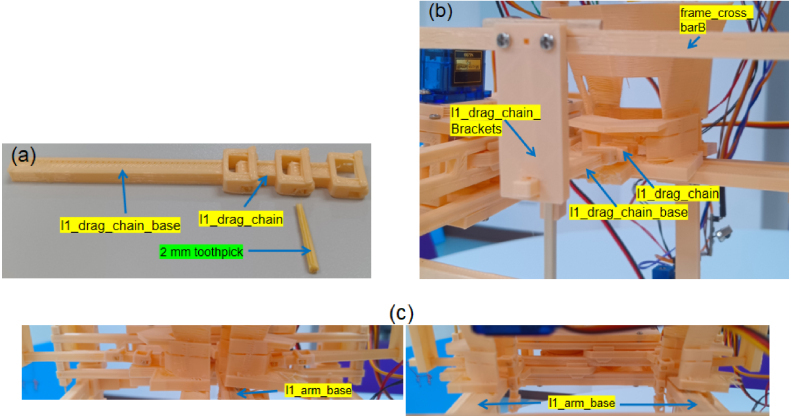
Assembly and operation of the shuttlecock head alignment chain: (a) Assembly of l1_drag_chain segments, (b) Assembled unit, where the position can be adjusted by sliding the drag chain base, and (c) Motion range of the base between the closed and open positions, optimized to allow proper rotation and downward settling of the shuttlecock head alignment.

• Fabricate all parts listed in Table 5.
• **Hopper:** Bond l1_hopper, l1_hopper_neck, and l1_hopper_flange. Mount onto l1_arm_base (Fig. 10(a)). Install l1_sensor_vertical_bar beneath the base with l1_sensor_housing attached.
• **Arm Guiding:** Install arm_end_plate_A and arm_end_plate_B (Fig. 10(b)). Add arm_guide_rail_B to constrain movement.
• **Alignment Chain:** Connect l1_drag_chain segments using 2 mm toothpicks. Sand joints to prevent friction. Attach to l1_drag_chain_base (Fig. 11(a)) and mount to l1_drag_chain_bracket on the frame (Fig. 11(b)). Fig. 11(c) shows the operational states.
• **Note:** Light sanding of sliding surfaces is crucial to minimize friction and prevent jamming. 6 × 2 mm wooden sticks can substitute arm_guide_rail_B.

**Table 6:** 3D-printed STL files and quantities used for the Layer 2 module.

STL File	Qty	STL File	Qty	STL File	Qty
l2_arm_base	2	l2_arm_base_key	2	arm_end_plate_B_l2	2
arm_guide_rail_B_l2	1	l2_housing	2	l2_latch	2
l2_lever	2	l2_pin	4	l2_pusher	2
l2_pusher_base	2	l2_spring_postA	2	l2_spring_postB	2

### 5.4 Layer 2: Pushing/separation

**Fig. 12:**
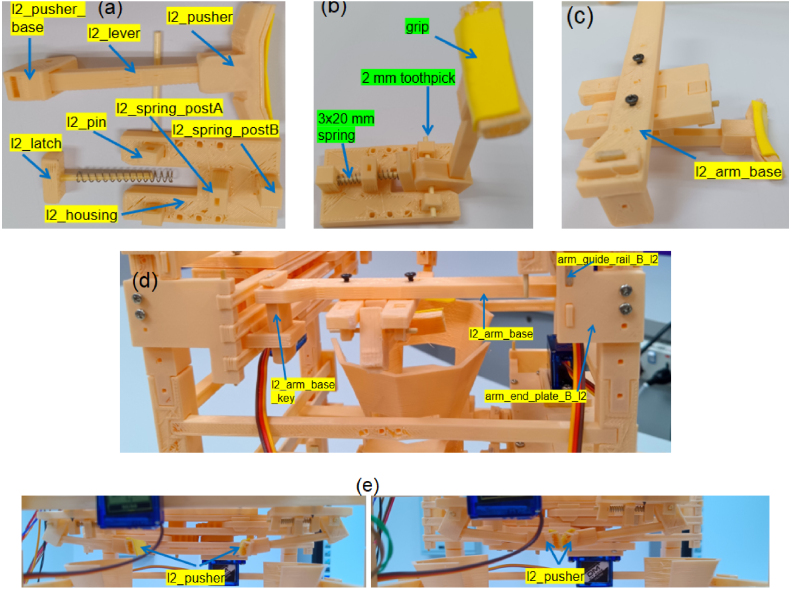
Layer 2 assembly: (a) Pusher and latch subassembly, (b) Completed pusher mechanism, (c) Installation on the Layer 2 arm base, (d) Integration of the module onto the main support frame, and (e) Motion range of the pusher showing the fully opened and closed positions.

• Fabricate all parts listed in Table 6 and the arm module (Table 4).
• **Subassembly:** Assemble pins and spring posts onto l2_housing. Combine l2_pusher, l2_lever, and l2_pusher_base. Bond l2_latch to a 2 mm toothpick (Fig. 12(a)).
• **Pusher Assembly:** Complete the structure (Fig. 12(b)). The l2_latch defines two stable positions (horizontal/vertical). Wrap the pusher tip with a badminton handle grip for friction. Lightly sand lower surfaces of the lever and pusher.
• **Installation:** Mount onto l2_arm_base (Fig. 12(c)) and integrate into the main frame (Fig. 12(d)). Verify the full motion range (Fig. 12(e)).

**Table 7:** 3D-printed STL files and quantities used for L3 module.

STL File	Qty	STL File	Qty	STL File	Qty
l3_gripper	2	l3_spacer	2	–	–

### 5.5 Layer 3: Gripping

**Fig. 13:**
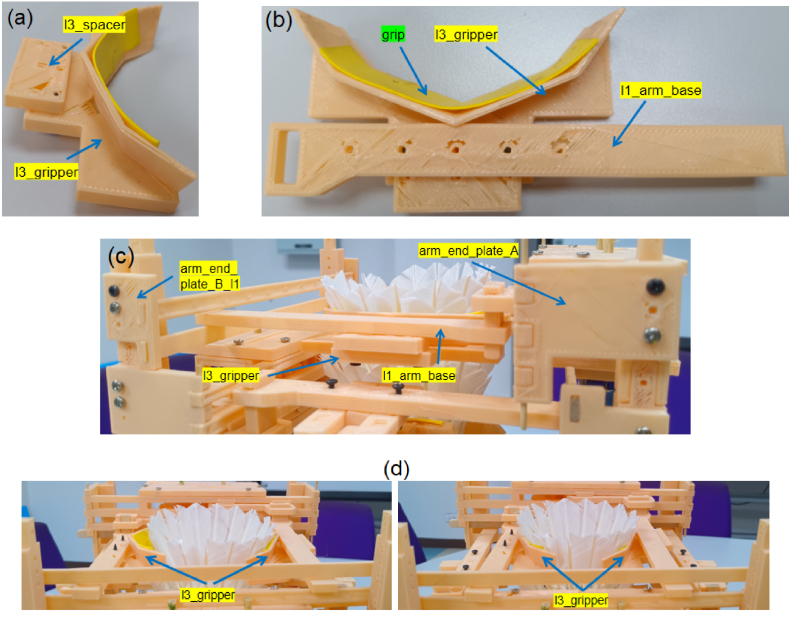
Layer 3 assembly: (a) Construction of the gripper with badminton grip, (b) Arm mounting of gripper and spacer components, (c) Integration of the module onto the main support frame, and (d) Gripper motion range.

• Fabricate parts in Table 7 and the arm module (Table 4).
• Wrap l3_gripper with badminton grip tape (Fig. 13(a)).
• Install l3_gripper and l3_spacer on the **left arm only** (Fig. 13(b)).
• Integrate onto the main frame (Fig. 13(c)) and verify motion (Fig. 13(d)).

### 5.6 Wiring configuration

**Fig. 14:**
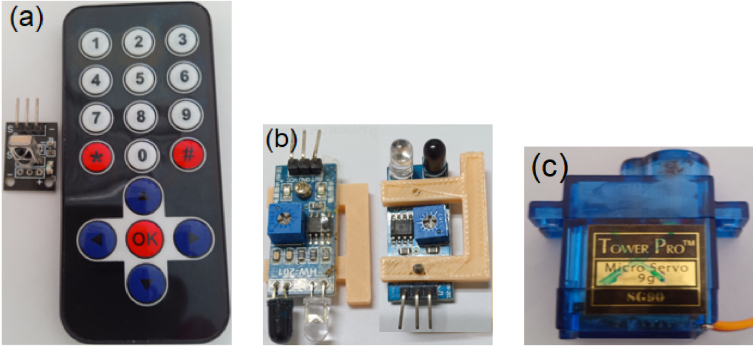
Peripheral modules used for control and sensing: (a) IR remote/receiver, (b) Obstruction sensor oriented for downward detection by bending the diode perpendicular to the circuit board, and (c) SG90 servo.

The system connects the Arduino Uno to the servos and sensors as detailed in Table 8. Peripheral components are shown in Fig. 14. Using a 5.0 V battery for the SG90 servos helps prevent voltage drops under load. It is crucial to share a common ground across all power supplies.

**Table 8:** Electrical wiring configuration between the Arduino Uno and system components.

Component	Pin	Function
Gate Servo	D9	Open/close outlet
Push Servo	D10	Push shuttlecock
Grip Servo	D11	Hold/release shuttlecock
IR Receiver	D2	Remote input (keys 1, 2, 4, 5)
Obstruction Sensor (User)	A1	Detect hand trigger
External Power Supply	5 V/GND	Shared with Arduino ground

### 5.7 Calibration guidelines

**Fig. 15:**
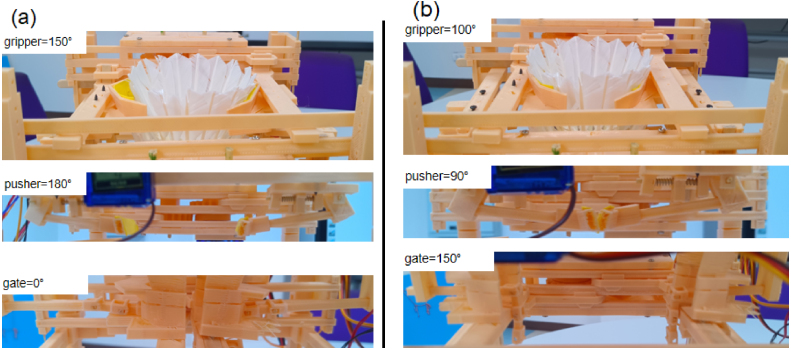
Servo calibration reference: (a) Closed configuration (initial state), and (b) Open configuration (dispensing state).

Reliable operation of the Grip-Gate-Push (GGP) mechanism requires proper calibration of the three servo stages. Fig. 15, Fig. 15 illustrate the closed (initial) and open (dispensing) positions. Use a slow-motion half-cycle (IR keys 4 and 5) to verify clearance and minimize friction.

**Closed State** (Fig. 15(a)):

• **Gripper: 150°** — Open without excessive compression.
• **Pusher: 180°** — Fully retracted (horizontal).
• **Gate: 0°** — Fully closed (supports stack).

**Open State** (Fig. 15(b)):

• **Gripper: 100°** — Moves forward to stabilize the second shuttlecock.
• **Pusher: 90°** — Rotates to vertical (non-obstructing).
• **Gate: 150°** — Fully open (dispensing).

### 5.8 Safety considerations

The ThongPaDisp prototype operates on a low-voltage 5 V DC supply; however, basic electrical and mechanical safety precautions should be observed during assembly and operation.

• **Electrical Safety:** All wiring connections should be properly insulated and secured to prevent short circuits. A stable 5 V power supply capable of delivering approximately 2 A is recommended to accommodate peak current draw during servo actuation. Insufficient current capacity may result in voltage drops or unstable microcontroller behavior.
• **Mechanical Clearance:** The Grip-Gate-Push mechanism includes moving actuating arms. Users should keep fingers of the dispensing outlet and internal shuttlecock column during operation to prevent minor pinching or mechanical interference.
• **Servo Calibration Limits:** Servo travel limits (minimum and maximum angles) must be calibrated carefully in firmware prior to operation. Driving a servo beyond its mechanical limits or against a jammed shuttlecock may cause prolonged stalling, leading to overheating and accelerated wear of internal plastic gears. Additionally, because the SG90 micro servos used in this prototype have limited torque, builders must precisely calibrate the firmware-defined travel limits to ensure that the push-head engages the shuttlecock at an angular position that maximizes mechanical advantage. Proper alignment ensures that peak torque is delivered at the point of maximum frictional resistance, which is critical for preventing jams and minimizing the need for multiple push cycles.
• **Operating Orientation:** The dispenser should be placed on a stable, level surface and maintained in a vertical orientation during use. Operating the device while tilted or unstable may lead to misalignment of the shuttlecock stack or unintended tipping of the unit.

## 6 Operation instructions

**Fig. 16:**
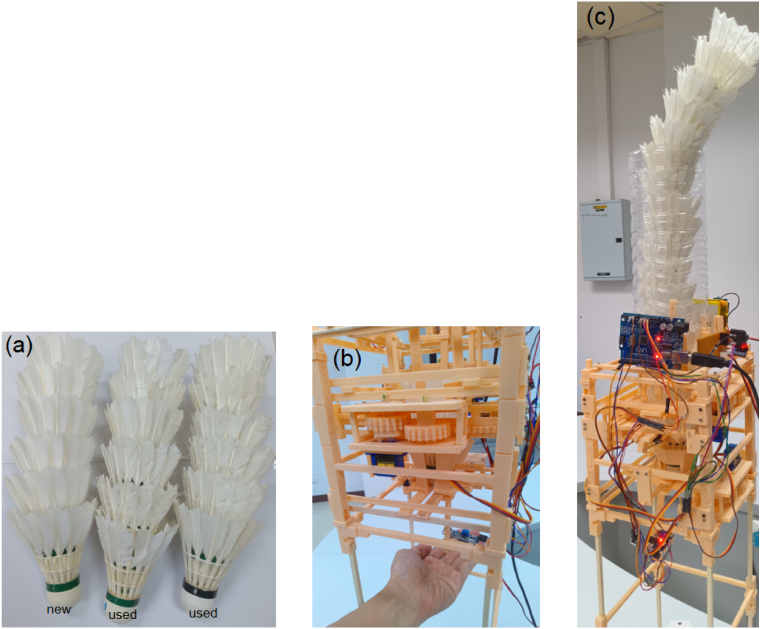
Shuttlecocks and experimental setup for the release-timing tests: (a) New and used feather shuttlecocks, (b) User hand placement beneath the prototype, and (c) ThongPaDisp prototype loaded with a vertical shuttlecock stack.

After assembling all mechanical components (Fig. 16(c)), prepare the system for operation as follows:

• **Controller and power installation:** Mount the Arduino Uno and the power supply on the top platform of the main support frame, as shown in Fig. 16(c). Route the electrical wiring from the top-mounted controller to the servo motors and sensors located in the lower layers of the device.
• **Electrical connections:** Connect all servo motors and sensors to the Arduino Uno according to the wiring assignments summarized in Table 8. Ensure that the power supply shares a common ground with the Arduino Uno, and that all signal and ground connections are securely attached.
• **Program upload:** Upload the control firmware (available in the repository) to the Arduino Uno via the USB interface.
• **Servo position verification:** Power on the system and verify that all three servo-driven layers (Layer 1 gating, Layer 2 pushing, and Layer 3 gripping) move to their default closed positions. Confirm that the closed and open configurations of each layer match the reference positions illustrated in Fig. 15.
• **Loading shuttlecocks:** Insert shuttlecocks through the top loading tube, which is fabricated from a modified plastic water bottle. Load the shuttlecocks one by one with the cork facing downward, ensuring that the stack is vertically aligned inside the tube.
• **Initial functional check:** Perform a single test cycle using the IR remote (Key 2) to verify smooth execution of the Grip-Gate-Push sequence without mechanical interference.

### 6.1 Operating modes

The ThongPaDisp system supports both automatic and manual operating modes.

#### 6.1.1 Hand-triggered automatic mode

In this mode, shuttlecock dispensing is triggered by the obstruction sensor mounted beneath the device. To request a shuttlecock, place a hand beneath the outlet opening. The triggering distance can be adjusted by configuring the obstruction sensor sensitivity. Once a hand is detected, the system automatically executes a single Grip-Gate-Push cycle and releases one shuttlecock, which drops directly into the user’s hand. After dispensing, the hand must be removed from the sensing area before another dispensing cycle can be initiated. Placing the hand beneath the device again triggers the next dispensing cycle.

#### 6.1.2 IR remote control mode

In addition to hand-triggered operation, the system supports wireless control via an infrared (IR) remote. This mode is primarily intended for testing, calibration, and debugging purposes. The key functions are defined as follows:

• **Key 1 (continuous mode):** Dispenses three shuttlecocks consecutively.
• **Key 2 (single shot):** Dispenses a single shuttlecock.
• **Key 4 (calibration – forward):** Executes the forward half-cycle, moving mechanisms to the open state. Useful for checking mechanical clearance or detecting jams.
• **Key 5 (calibration – return):** Executes the return half-cycle, resetting mechanisms to the closed/initial state.

## 7 Validation and characterization

### 7.1 Test preparation

The ThongPaDisp prototype weighs approximately 450 g. The 3D-printed structure weighs 313 g, the Arduino Uno controller weighs 25 g, the three SG90 servos weigh 27 g in total, and the battery pack weighs 85 g. A breakdown of the device weight is summarized in Table 10. A total of 18 shuttlecocks were used for validation, consisting of 6 new units and 12 used units (Fig. 16(a)). The new set consisted of Victor Champion No. 1 shuttlecocks in new condition, unused in court play and exhibiting zero feather breakage. To improve generalizability, the used set included both Victor Champion No. 1 and Venson A7 shuttlecocks. All used samples had undergone more than one year of regular court play and were selected according to a reproducible wear rubric summarized in Table 9. All shuttlecocks were loaded into the vertical column in a fixed sequence as shown in Fig. 16(c).

Because the overall dataset comprises 90 trials generated by repeatedly dispensing the same 18 shuttlecocks across five rounds, the data follows a repeated-measures structure in which observations are clustered by shuttlecock index. Consequently, individual dispensing events are not strictly independent. To reflect this structure, performance metrics were summarized at the shuttlecock-index level to characterize inter-index variability rather than treating all trials as independent samples. The experimental protocol was designed primarily to evaluate operational feasibility, repeatability, and jamming mitigation under representative training conditions. The objective was functional validation of the dispensing mechanism rather than a statistically powered inferential comparison between modes.

**Table 9:** Shuttlecock conditions and selection criteria used for experimental validation.

Condition	Brand	Objective selection criteria
New	Victor Champion No. 1	0 broken feathers; no visible fraying; unused in court play.

Used	Victor Champion No. 1, Venson A7	≥1 completely missing feather or ≥80% of feather tips visibly frayed.

**Fig. 17:**
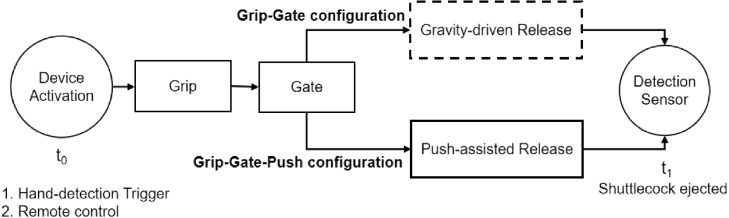
Schematic comparison of the dispensing sequence between the Grip-Gate (gravity-driven release) and Grip-Gate-Push (push-assisted release) configurations.

Timing measurements were obtained by logging timestamps on the Arduino using the millis() function. As shown in Fig. 17, a start event ($t_{0}$) was registered when the user-trigger sensor detected a hand placed beneath the device (Fig. 16(b)), and a stop event ($t_{1}$) was recorded when the falling shuttlecock crossed the lower optical sensor positioned 7 cm below the outlet. This setup captured the complete dispensing interval for both the Grip-Gate and Grip-Gate-Push configurations, allowing for a direct comparison of release reliability and timing dynamics.

**Table 10:** Weight breakdown of the complete ThongPaDisp prototype.

Component	Weight (g)
3D-printed structure	313
Arduino Uno	25
Servo motors (3× SG90)	27

**Subtotal (device only)**	**365**
Battery pack	85

**Total Operational Weight**	**450**

### 7.2 Grip-Gate (gravity) test

The first experiment evaluated the performance of passive shuttlecock dispensing using only the Grip-Gate subsystem, without push assistance. Five experimental repetitions were conducted. In each repetition, the same ordered sequence of 18 shuttlecocks (6 new followed by 12 used) was tested. Each shuttlecock was labeled with a fixed index to enable tracking of repeated jamming behavior over five runs.

#### 7.2.1 Jamming incidence analysis

A jamming event was defined as the shuttlecock failing to pass through the gate after actuation, requiring manual intervention. Table 11 summarizes the indices at which jamming occurred over the five repetitions. New shuttlecocks successfully passed through the gate in all but one case, corresponding to a single jam at index 4 during Round 4. In contrast, used shuttlecocks exhibited frequent jamming, often recurring at the same indices (3, 5, 7, 9, and 10), suggesting a strong correlation with feather deformation patterns.

Across five repetitions per shuttlecock index, a total of 30 dispensing events were recorded for new shuttlecocks (6 indices × 5 trials) and 60 events for used shuttlecocks (12 indices × 5 trials). At the trial level, one jamming event occurred among new shuttlecocks (3.3%), whereas 24 jamming events were observed among used shuttlecocks (40.0%). Because each shuttlecock was evaluated repeatedly, jamming proportions were aggregated at the shuttlecock-index level to account for inter-index variability. The 95% confidence intervals (CI) were calculated using the standard formula for the mean: $CI=\overset{¯}{p}\pm t_{\alpha /2,df}\left(s/\sqrt{N}\right)$, where $\overset{¯}{p}$ is the mean proportion, s is the inter-index standard deviation, N is the number of indices, and t is the critical value from Student’s t-distribution.

For used shuttlecocks (N=12 indices), the mean index-level failure proportion was $\overset{¯}{p}=0.400$ with an inter-index standard deviation of s=0.443. Using the critical value $t_{0.025,11}=2.201$, the 95% CI was estimated as [0.119,0.682]. For new shuttlecocks (N=6 indices), the mean proportion was $\overset{¯}{p}=0.033$ with a standard deviation of s=0.082. Using $t_{0.025,5}=2.571$, the calculated interval was [−0.053,0.119], which is constrained to [0,0.119].

**Table 11:** Shuttlecock jamming occurrences by index recorded over five Grip-Gate (gravity-only) test repetitions.

Group	R1	R2	R3	R4	R5
New Jamming occurrences	–	–	–	4	–
Used Jamming occurrences	3,5,7,9	3,5,6,7,9,10	3,5,7,9,10	1,3,5,7,10	3,5,7,9

#### 7.2.2 Fall-time measurement

Time-to-sensor data were recorded for all successful drops. Summary statistics are reported in Table 12. New shuttlecocks exhibited a tightly clustered mean fall time of approximately 0.28 s with low variance. Used shuttlecocks showed a similar mean fall time but with substantially greater dispersion, including occasional prolonged descents. This increased variability further reflects the influence of feather wear on gravity-driven release behavior.

**Video demonstration:** A recording of the Grip–Gate test is available on Zenodo at: https://zenodo.org/records/18899461

**Table 12:** Summary of jamming counts and fall-time statistics for Grip-Gate (gravity-only) operation over five test repetitions.

Condition	R1	R2	R3	R4	R5	Total Jams (n/N)	Jamming Rate [95% CI]	Mean ± SD (s)	Min–Max (s)
New	0	0	0	1	0	1/30	3.3% [0.0%, 11.9%]	0.284 ± 0.013	0.268–0.325
Used	4	6	5	5	4	24/60	40.0% [11.9%, 68.2%]	0.287 ± 0.035	0.258–0.468

### 7.3 Grip-Gate-Push test

The second experiment evaluated the full Grip-Gate-Push (GGP) dispensing sequence. Five experimental repetitions were conducted using the same shuttlecock set and index order as in the gravity-only test, resulting in a total of 90 dispensing events. In the GGP configuration, a failure event was defined as a shuttlecock requiring more than one push cycle to be successfully dispensed. Under this criterion, all new shuttlecocks were released on the first push attempt. Among used shuttlecocks, a single event (index 5 in Round 1) required three push cycles due to contact interference with the shuttlecock above it. Despite the delay, the system completed the dispensing process autonomously without manual intervention.

Because the same shuttlecock indices were evaluated in both test modes, the identical cluster-aware statistical approach was applied. For used shuttlecocks (N=12 indices), the active GGP mechanism dramatically reduced the mean index-level failure proportion to $\overset{¯}{p}=0.017$ with an inter-index standard deviation of s=0.058. Using the critical value $t_{0.025,11}=2.201$, the calculated interval was [−0.020,0.054], which is constrained to [0,0.054]. For new shuttlecocks (N=6 indices), zero jamming events were recorded across all repetitions, yielding a mean proportion of $\overset{¯}{p}=0.000$ with a standard deviation of s=0.000. Using $t_{0.025,5}=2.571$, the calculated interval was [0,0].

Release-time statistics for the GGP mode are summarized in Table 13. New shuttlecocks exhibited a mean release time of 0.62 s, while used shuttlecocks exhibited a comparable mean release time of 0.61 s. The low failure rate and consistent release times demonstrate that the addition of an active push stage substantially improves dispensing reliability compared to gravity-only operation.

**Table 13:** Push-attempt counts and release-time statistics for Grip-Gate-Push operation over five test repetitions.

Condition	R1	R2	R3	R4	R5	>1 Push (n/N)	>1 Push Rate [95% CI]	Mean ± SD (s)	Min–Max (s)
New	0	0	0	0	0	0/30	0.0% [0.0%, 0.0%]	0.619 ± 0.023	0.590–0.675
Used	1	0	0	0	0	1/60	1.7% [0.0%, 5.4%]	0.611 ± 0.022	0.577–0.682

#### 7.3.1 Timing characteristics of used shuttlecocks

To examine whether shuttlecocks that frequently jammed in gravity-only mode exhibited distinct behavior under GGP operation, the used shuttlecocks were divided into two groups based on their gravity-test performance. The first group consisted of indices with high jamming frequency (indices 3, 5, 7, 9, and 10), while the second group included indices with low or no jamming occurrences.

**Fig. 18:**
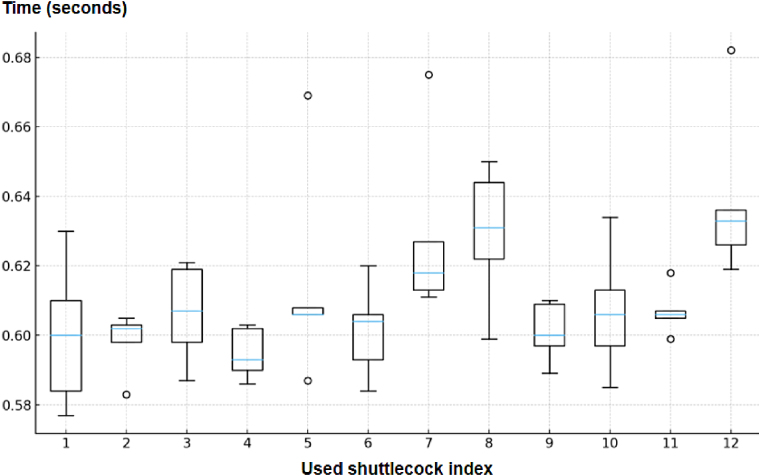
Boxplot of release time distribution for used shuttlecocks over five repetitions.

Release-time statistics for these two groups are compared in Table 14. Shuttlecocks belonging to the high-jamming group exhibited a slightly higher mean release time and greater variance than the remaining indices. Index 12 was excluded from the timing analysis because it was positioned at the top of the vertical stack and therefore did not experience the same contact and loading conditions as the other shuttlecocks. Fig. 18 illustrates this difference through boxplot visualization. Although the GGP mechanism successfully prevented jamming, feather deformation continued to influence dynamic release behavior, leading to broader timing distributions for heavily worn shuttlecocks.

**Table 14:** Comparison of release-time statistics for used shuttlecock indices in Grip-Gate-Push operation.

Group	Mean (s)	SD (s)
High-stuck (3, 5, 7, 9, 10)	0.612	0.020
Other indices (1, 2, 4, 6, 8, 11)	0.605	0.011

**Fig. 19:**
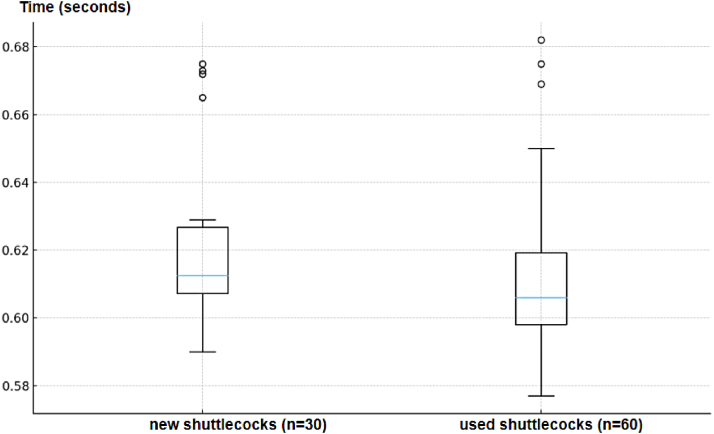
Comparison of release-time distribution for new and used shuttlecocks over five Grip-Gate-Push repetitions.

#### 7.3.2 New vs used timing distribution

Fig. 19 compares the overall release-time distributions for new and used shuttlecocks across all five GGP repetitions. While the mean release times are similar, the used shuttlecocks show a clearly broader dispersion. Conversely, new shuttlecocks tend to exhibit slightly higher mean release times, which may be attributed to their fuller, more intact feather structure, which increases aerodynamic drag and provides a larger surface area for reactive contact within the mechanism.

**Video demonstration:** A recording of the Grip–Gate–Push test is available on Zenodo at: https://zenodo.org/records/18899461

### 7.4 Comparison between dispensing modes

A direct comparison between Grip-Gate and Grip-Gate-Push operation is summarized in Table 15 . In gravity-only mode, repeated jamming was observed at specific indices, particularly among used shuttlecocks. In contrast, the GGP mode successfully dispensed all shuttlecocks over all test repetitions, with only one instance requiring multiple push cycles. While the mean release time increased from approximately 0.28 s in gravity-only operation to approximately 0.61 s in GGP operation, the increase reflects the additional mechanical stages required for active separation. This trade-off shows that improved dispensing reliability comes at the expense of longer dispensing cycles.

**Table 15:** Performance comparison between Grip-Gate and Grip-Gate-Push dispensing modes over five experimental repetitions.

	New Shuttlecock Index						Used Shuttlecock Index											
	1	2	3	4	5	6	1	2	3	4	5	6	7	8	9	10	11	12
(a) Jamming Count (GG)	0	0	0	**1**	0	0	**1**	0	**5**	0	**5**	**1**	**5**	0	**4**	**3**	0	0
(b) Mean Fall Time (s) (GG)	0.286	0.279	0.278	0.294	0.287	0.280	0.348	0.275	–	0.285	–	0.277	–	0.289	**0.283**	**0.269**	0.265	0.286

(c) Push-Attempt Count (>1) (GGP)	0	0	0	**0**	0	0	**0**	0	**0**	0	**1**	**0**	**0**	0	**0**	**0**	0	0
(d) Mean Release Time (s) (GGP)	0.621	0.620	0.616	0.615	0.626	0.619	0.600	0.598	**0.606**	0.595	**0.615**	0.601	**0.629**	0.629	**0.601**	**0.607**	0.607	0.639

### 7.5 Power consumption

To evaluate the suitability of the system for portable operation, empirical power consumption measurements were conducted. The electrical behavior of the ThongPaDisp prototype can be separated into a continuous monitoring phase and a transient actuation phase. The results are summarized in Table 16.

During operation, the Arduino controller continuously monitors the infrared sensors and the hand-trigger input. The baseline current draw of the controller was measured at approximately 0.05 A (50 mA). Over the 9-minute experimental run used for the dispensing tests, this monitoring load corresponded to an energy consumption of approximately 7 mAh. During the same experiment, a total of 90 shuttlecocks were dispensed. The three SG90 servo actuators consumed approximately 8 mAh in total during these 90 dispensing cycles. The maximum transient current observed during actuation was approximately 0.6 A when multiple servos were active simultaneously.

**Table 16:** Measured electrical characteristics of the ThongPaDisp prototype during a 9-minute dispensing test (90 cycles). Arduino current represents continuous operation, while servo current indicates the peak transient current during actuation.

Subsystem	Operating Mode	Measured Peak	Energy Consumption
		Current (mA)	(mAh)
Arduino Uno	Continuous monitoring (9 min)	∼50	7
Servos (3× SG90)	Dispensing operation (90 cycles)	∼600	8

**Total**	9-minute dispensing test (90 cycles)	–	**15**

## 8 Discussion

The experimental results demonstrate that shuttlecock condition plays a critical role in dispensing reliability, particularly in systems that rely solely on passive gravity. In the Grip-Gate (gravity-only) tests, new shuttlecocks were released successfully in nearly all trials, whereas used shuttlecocks exhibited frequent and recurring jamming. These jamming events were concentrated at specific indices, indicating that feather deformation and asymmetry introduce consistent frictional and geometric interference within the vertical column. This behavior highlights a fundamental limitation of gravity-driven dispensing when handling deformable, irregularly shaped objects.

In contrast, the full Grip-Gate-Push (GGP) mechanism successfully dispensed all shuttlecocks across the experimental evaluation without requiring manual intervention. The addition of an active push stage provided sufficient mechanical force to overcome friction and contact interference caused by feather wear and interlocking. Although one used shuttlecock required multiple push cycles, the system remained capable of completing the dispensing sequence autonomously. These results indicate that active separation significantly improves robustness against variations in shuttlecock condition, while preserving consistent operational behavior. This finding indicates that actuator selection and push-head geometry play important roles in defining the system’s force margin and may influence long-term reliability under sustained use with worn shuttlecocks. In the current prototype, the firmware operates in an event-driven manner in which each dispensing cycle is triggered by either the infrared sensor or the IR remote command and the system returns to an idle monitoring state after completion, thereby preventing uncontrolled repetitive actuation if a mechanical anomaly occurs.

Despite the elimination of jamming events in GGP mode, timing analysis revealed that shuttlecocks with high jamming frequency in gravity-only operation continued to exhibit larger release-time variability. This observation suggests that while the push stage ensures reliable separation, it does not fully homogenize the release dynamics. Feather deformation continues to influence contact interactions during actuation, leading to greater dispersion in release timing for heavily worn shuttlecocks. Consequently, shuttlecock condition remains a contributing factor to dynamic behavior even in actively assisted systems.

A trade-off between dispensing reliability and cycle time was also observed. Gravity-only operation yielded shorter mean fall times, whereas the GGP sequence approximately doubled the total release duration due to the additional mechanical stages. From a practical training perspective, this increase represents an acceptable compromise, as consistent and uninterrupted shuttlecock delivery is generally prioritized over shorter cycle times. The predictable timing of the GGP sequence further supports its suitability for repetitive training drills.

To contextualize the observed cycle time within the broader landscape of training equipment, commercially available shuttlecock feeders such as the Siboasi SS-B7 (advertised serving frequency 0.75–7 s per shuttlecock) and the SS-B2300 A (1.2–5.5 s) report total cycle times that encompass separation, positioning, and launching [11], [16]. While the internal timing distribution between singulation and propulsion stages is not publicly disclosed, the advertised serving frequency provides an indicative reference for overall system throughput in commercial devices.

As summarized in Table 17, ThongPaDisp differs architecturally by isolating and evaluating the singulation stage independently from propulsion. The measured 0.61 s release time therefore represents only the separation phase and is not directly comparable to integrated commercial cycle times. Assuming a symmetric actuation profile in which the auxiliary phases, such as closing and opening motions (Fig. 15), require a duration comparable to the measured release phase, the total mechanical cycle time can be approximated as ∼1.22 s. This value represents an estimate rather than a directly measured total cycle duration. Within this context, the observed cadence indicates that the proposed Grip-Gate-Push mechanism operates within the lower bound of advertised commercial serving intervals.

**Table 17:** Contextual Comparison of representative Shuttlecock dispensing systems.

System	Dispensing Architecture	Serving Frequency	Open-Source
Siboasi SS-B7 [11]	Grip-Prepare-Release	0.75–7.0 s (total cycle)	No
Siboasi SS-B2300A [16]	–	1.2–5.5 s (total cycle)	No
ThongPaDisp (this work)	Grip-Gate-Push	0.61 s (measured release time);	Yes
		∼1.22 s (estimated total cycle)	

### 8.1 Mechanism-level comparison with existing approaches

Shuttlecock dispensing mechanisms may be conceptually contrasted as “Grip-Prepare-Release” and “Grip-Gate-Push” architectures. In commercial systems such as the Siboasi SS-B7 [11], the dispensing process can be described as a “Grip-Prepare-Release” sequence. Paired gripping elements isolate the lowest shuttlecock from the stack, adjust their aperture to accommodate alignment, and subsequently release the shuttlecock toward the launching module.

In contrast, the proposed Grip-Gate-Push (GGP) mechanism separates the dispensing process into three functional layers: stabilization, release, and active pushing. The gripper stabilizes the shuttlecock stack, the gate regulates single-unit release, and the pusher actively separates the lowest shuttlecock. This three-layer decomposition enables explicit control of the separation process while maintaining a modular structure suitable for open-source fabrication.

### 8.2 Mechanical robustness and failure analysis

While the Grip-Gate-Push (GGP) mechanism demonstrated high reliability during short-term trials, understanding its long-term mechanical robustness is important for practical deployment.

1. **Servo Gear Degradation** The system employs low-cost plastic-gear servo motors. During extended operation, repeated actuation under varying load conditions may lead to gradual gear wear or torque loss within the servo gearbox.
2. **Joint Deformation at Force Transmission Interfaces** Mechanical joints that transmit actuation forces, particularly the connection between the rack mechanism and the actuator arms, are subject to repeated loading. These joints experience resistive forces during gripping, pushing, and gating operations. Over prolonged use, insufficient structural stiffness may result in minor deformation or bending. To increase stiffness in these regions, reinforcement using thin wooden sticks can be incorporated as an additional structural support.
3. **Sliding Friction in Moving Components** Several components rely on sliding motion and are fabricated from PLA+. Surface roughness inherent to FDM printing can increase friction and mechanical resistance during operation. Post-processing by light sanding of sliding surfaces can reduce friction and help maintain consistent actuator performance over time.

**Modularity Considerations:** The current design intentionally employs multiple smaller printed components rather than a small number of large integrated parts. While larger monolithic prints could potentially reduce assembly steps and increase structural rigidity, the modular architecture was deliberately selected to improve long-term maintainability. This approach reduces print failure risk during fabrication, minimizes material waste, and enables rapid, low-cost replacement of individual high-wear components without requiring reprinting of entire subassemblies.

**Expected Lifetime and Maintenance:** The service life of printed components depends on operating frequency, environmental conditions, and shuttlecock deformation characteristics. Although long-term endurance testing was beyond the scope of the present study, the most wear-prone components are expected to be the rack–pinion interface and the sliding tracks associated with the pusher mechanism. A key advantage of the open-source hardware architecture is maintainability: individual components such as the pinion gear, gripper arms, or sliding guides can be rapidly reprinted and replaced at minimal cost. This modular replaceability enables practical long-term operation even when wear-related degradation occurs.

### 8.3 Energy consumption and battery runtime

Based on the measured electrical characteristics, the servo actuators consumed approximately 8 mAh during the 90-cycle dispensing experiment, while the Arduino controller consumed approximately 7 mAh during the same 9-minute operating period. The total system energy consumption during the experiment was therefore approximately 15 mAh.

This corresponds to an average system energy consumption of approximately 0.17 mAh per dispensing cycle. Assuming a portable battery pack with a nominal capacity of 2000 mAh, the system could theoretically support approximately 10,000 dispensing cycles under similar operating conditions. In practice, the achievable number of cycles may be lower depending on the duty cycle, battery conversion efficiency, and additional electronic overhead.

## 9 Limitations and future improvements

### 9.1 Orientation sensitivity

Although ThongPaDisp demonstrated reliable operation under controlled experimental conditions, several limitations were identified during testing. First, the current system performs optimally when installed in a fully upright orientation. When the device is tilted, shuttlecocks may shift within the hopper, leading to misalignment, unreliable sensor triggering, or incomplete engagement with the gating mechanism. As a result, the present design is best suited for use on flat, level surfaces such as tables or fixed training stands.

### 9.2 Material durability

Material selection also represents a practical limitation for long-term deployment. While PLA+ was selected for the structural components due to its accessibility, cost-effectiveness, and ease of printing, it presents challenges for extended use. PLA-based materials are susceptible to long-term creep deformation, wear, or fracture under continuous mechanical loading. Furthermore, PLA possesses a relatively low glass transition temperature (approximately 60 °C). In practical scenarios, such as extended use in high-cycle training environments, unconditioned badminton halls, or storage in hot vehicles, structural warping may occur over time. For applications requiring improved thermal and mechanical stability, components may be fabricated using alternative thermoplastics such as PETG or ABS.

### 9.3 Actuator force margin and stack load

The storage tube, constructed by repurposing a standard 1.5 L plastic water bottle (approximately 22 cm in height), accommodates a stack of 12 feather shuttlecocks and can hold up to 18 shuttlecocks without lateral spillage. Assuming an average mass of approximately 5 g per shuttlecock, the total vertical load ranges from 60 g to 90 g. Previous empirical characterization of a similar 3D-printed shuttlecock manipulation mechanism reported a resistive force of approximately 200 g during shuttlecock separation [15]. Experimental observations from the present prototype are consistent with this range, confirming that the actuator arms provide sufficient mechanical capacity to reliably support the current vertical load (60–90 g).

Consequently, the maximum shuttlecock capacity of the present prototype is not limited by the holding force of the actuators. Based on the reported force margin, the actuator mechanism could theoretically support a substantially larger stack of shuttlecocks. In the current design, the practical capacity is instead constrained by the physical height of the selected storage tube.

However, actuator performance introduces an additional dynamic constraint. While the SG90 micro servo motors provide sufficient torque for standard dispensing operations, the observed multi-push event involving a heavily deformed shuttlecock suggests that the available force margin may become limited under extreme geometric interference conditions. Upgrading to higher-torque or metal-gear servos, or optimizing the push-head geometry to reduce contact resistance, could further improve robustness when handling severely worn shuttlecocks.

### 9.4 Long-term endurance considerations

To better characterize long-term durability, future work could implement a structured endurance evaluation protocol. Such testing may involve repeated dispensing cycles under controlled conditions (e.g., several thousand cycles) to systematically observe mechanical wear and actuator performance over time. During extended operation, monitoring indicators such as actuation consistency, abnormal noise, or increased mechanical resistance may help identify early signs of component fatigue.

### 9.5 Adaptability and application extensions

Beyond its present application, the modular Grip–Gate–Push architecture can be adapted for different shuttlecock geometries, including synthetic nylon or hybrid variants. Moreover, the principle of staged mechanical separation may be extended to other stacked, conical, or friction-prone objects, such as nested paper cups.

### 9.6 Protective enclosure and cable management

A lightweight protective enclosure could help shield internal components while maintaining operational accessibility. In conjunction with this enclosure, the electronics mounting and cable routing could be structurally integrated into the frame design to reduce wire clutter and improve overall system safety and robustness.

## 10 Conclusion

This study presented ThongPaDisp, a lightweight and low-cost shuttlecock dispenser developed as an open-source hardware platform based on a three-stage Grip-Gate-Push (GGP) dispensing architecture. The GGP mechanism implemented in ThongPaDisp was specifically designed to overcome the limitations of conventional gravity-only (GG) dispensing systems, which are prone to high jamming rates when handling used shuttlecocks due to feather deformation and interlocking within the stack. By incorporating an active push stage, the GGP mechanism substantially improved dispensing reliability, enabling reliable release of both new and used shuttlecocks during repetitive training sessions.

The system is modular and reproducible using consumer-grade 3D printing, with all design files, firmware, and assembly documentation made openly available. Dual operating modes, automatic hand-triggered dispensing and manual infrared control, provide flexibility for both practical training use and controlled experimentation. Current limitations include the long-term durability of PLA+ structural components, the torque margin of plastic-gear servo motors under extreme deformation conditions, and sensitivity to device orientation. Future improvements may include higher-torque or metal-gear servos, optimized push-head geometry, alternative load-bearing materials, and optional directional release mechanisms to support more dynamic training scenarios.

## CRediT authorship contribution statement

**Thanawat Saeeab:** Writing – original draft, Visualization, Software, Resources, Project administration, Methodology, Investigation, Conceptualization. **Wichuda Phiphitphibunsuk:** Writing – review & editing, Visualization, Validation, Methodology, Investigation, Formal analysis, Conceptualization.

## Ethics statement

This work did not involve human participants, animal subjects, or any procedures requiring ethical approval.

## IP disclosure

Thai Patent Application Nos. 2101004228 A and 2401000673 A have been published for advertisement and are owned by the University of Phayao. In addition, Thai Patent Application No. 2501004788 has been filed and is currently pending publication. The inventors and rights holders have explicitly agreed to release the disclosed mechanisms under the CERN Open Hardware Licence Version 2 - Strongly Reciprocal (CERN-OHL-S v2) in compliance with open-source hardware standards.

## Declaration of competing interest

The authors declare that they have no known competing financial interests or personal relationships that could have appeared to influence the work reported in this paper.

## Appendix A Supplementary data

MMC S1 3D-printable STL files and Arduino firmware for the ThongPaDisp system, hosted on Zenodo. MMC S1

## Footnotes

1 The Arduino firmware source code is available at https://zenodo.org/records/18899461/files/a09ThongPaDisp_1_03_arduino_code.ino.
