# Supplementary material for: ThongPaDisp: An open-source 3D-printed shuttlecock dispenser using a Grip-Gate-Push mechanism
Source: HardwareX. 2026 Apr 29;26:e00783. doi: 10.1016/j.ohx.2026.e00783 (PMC13157078; doi:10.1016/j.ohx.2026.e00783)
Supplement: MMC S1 — 3D-printable STL files and Arduino firmware for the ThongPaDisp system, hosted on Zenodo. [file mmc1.pdf]

Table S1: Frame structure design files

Design filename	File type	Open source license	Location of the file
frame_base_beam	STL	CERN-OHL-S v2	https://zenodo.org/records/18899461/files/frame_base_beam.stl
frame_corner_bottomA	STL	CERN-OHL-S v2	https://zenodo.org/records/18899461/files/frame_corner_buttomA.stl
frame_corner_bottomB	STL	CERN-OHL-S v2	https://zenodo.org/records/18899461/files/frame_corner_buttomB.stl
frame_corner_post_bracket	STL	CERN-OHL-S v2	https://zenodo.org/records/18899461/files/frame_corner_post_bracket.stl
frame_corner_post_spacerA	STL	CERN-OHL-S v2	https://zenodo.org/records/18899461/files/frame_corner_post_spacerA.stl
frame_corner_post_spacerB	STL	CERN-OHL-S v2	https://zenodo.org/records/18899461/files/frame_corner_post_spacerB.stl
frame_cross_barB	STL	CERN-OHL-S v2	https://zenodo.org/records/18899461/files/frame_cross_barB.stl
frame_post_extA1	STL	CERN-OHL-S v2	https://zenodo.org/records/18899461/files/frame_post_extA1.stl
frame_post_extA2	STL	CERN-OHL-S v2	https://zenodo.org/records/18899461/files/frame_post_extA2.stl
frame_cross_barC**	STL	CERN-OHL-S v2	https://zenodo.org/records/18899461/files/frame_cross_barC1.stl or C2.stl
frame_top_barA2	STL	CERN-OHL-S v2	https://zenodo.org/records/18899461/files/frame_top_barA2.stl
frame_top_barB	STL	CERN-OHL-S v2	https://zenodo.org/records/18899461/files/frame_top_barB.stl
frame_l-bracketA	STL	CERN-OHL-S v2	https://zenodo.org/records/18899461/files/frame_l-bracketA.stl
frame_l-bracketD**	STL	CERN-OHL-S v2	https://zenodo.org/records/18899461/files/frame_l-bracketD1.stl or D2.stl
frame_l-bracketB	STL	CERN-OHL-S v2	https://zenodo.org/records/18899461/files/frame_l-bracketB.stl
frame_l-bracketC	STL	CERN-OHL-S v2	https://zenodo.org/records/18899461/files/frame_l-bracketC.stl
frame_l-bracketE	STL	CERN-OHL-S v2	https://zenodo.org/records/18899461/files/frame_l-bracketE.stl
frame_l-bracketG**	STL	CERN-OHL-S v2	https://zenodo.org/records/18899461/files/frame_l-bracketG1.stl
frame_post_extB1	STL	CERN-OHL-S v2	https://zenodo.org/records/18899461/files/frame_post_extB1.stl
frame_post_extB2	STL	CERN-OHL-S v2	https://zenodo.org/records/18899461/files/frame_post_extB2.stl
frame_cross_barA	STL	CERN-OHL-S v2	https://zenodo.org/records/18899461/files/frame_cross_barA.stl
frame_post_clipA	STL	CERN-OHL-S v2	https://zenodo.org/records/18899461/files/frame_post_clipA.stl

Table S2: Arm drive and common motion component design files

Design filename	File type	Open source license	Location of the file
arm_base_plate	STL	CERN-OHL-S v2	https://zenodo.org/records/18899461/files/arm_base_plate.stl
arm_end_plate_A	STL	CERN-OHL-S v2	https://zenodo.org/records/18899461/files/arm_end_plateA.stl
arm_end_plate_B_l1	STL	CERN-OHL-S v2	https://zenodo.org/records/18899461/files/arm_end_plate_B_l1.stl
arm_end_plate_B_l2	STL	CERN-OHL-S v2	https://zenodo.org/records/18899461/files/arm_end_plate_B_l2.stl
arm_spacer_plate	STL	CERN-OHL-S v2	https://zenodo.org/records/18899461/files/arm_spacer_plate.stl
arm_gear_shaftA	STL	CERN-OHL-S v2	https://zenodo.org/records/18899461/files/arm_gear_shaftA.stl
arm_gear_shaftB	STL	CERN-OHL-S v2	https://zenodo.org/records/18899461/files/arm_gear_shaftB.stl
arm_guide_rail_A	STL	CERN-OHL-S v2	https://zenodo.org/records/18899461/files/arm_guide_railA.stl
arm_guide_rail_B	STL	CERN-OHL-S v2	https://zenodo.org/records/18899461/files/arm_guide_railB.stl
arm_guide_rail_B_l2	STL	CERN-OHL-S v2	https://zenodo.org/records/18899461/files/arm_guide_rail_B_l2.stl
arm_pan_bracket	STL	CERN-OHL-S v2	https://zenodo.org/records/18899461/files/arm_pan_bracket.stl
arm_plate_guide_rod	STL	CERN-OHL-S v2	https://zenodo.org/records/18899461/files/arm_plate_guide_rod.stl
arm_servo_housing	STL	CERN-OHL-S v2	https://zenodo.org/records/18899461/files/arm_servo_housing.stl
arm_pinion	STL	CERN-OHL-S v2	https://zenodo.org/records/18899461/files/arm_pinion.stl
arm_rackL	STL	CERN-OHL-S v2	https://zenodo.org/records/18899461/files/arm_rackL.stl
arm_rackR	STL	CERN-OHL-S v2	https://zenodo.org/records/18899461/files/arm_rackR.stl
arm_rack_support	STL	CERN-OHL-S v2	https://zenodo.org/records/18899461/files/arm_rack_support.stl

Table S3: Layer 1 (gating and sensing) design files

**Design filename**	**File type**	**Open source license**	**Location of the file**
l1_arm_base_key**	STL	CERN-OHL-S v2	https://zenodo.org/records/18899461/files/l1_arm_base_keyA.stl or B.stl
l1_arm_base**	STL	CERN-OHL-S v2	https://zenodo.org/records/18899461/files/l1_arm_baseA.stl or B.stl
l1_hopper_flange	STL	CERN-OHL-S v2	https://zenodo.org/records/18899461/files/l1_hopper_flange.stl
l1_hopper_neck	STL	CERN-OHL-S v2	https://zenodo.org/records/18899461/files/l1_hopper_neck.stl
l1_hopper	STL	CERN-OHL-S v2	https://zenodo.org/records/18899461/files/l1_hopper.stl
l1_drag_chain	STL	CERN-OHL-S v2	https://zenodo.org/records/18899461/files/l1_drag_chain.stl
l1_drag_chain_base	STL	CERN-OHL-S v2	https://zenodo.org/records/18899461/files/l1_drag_chain_base.stl
l1_drag_chain_bracket	STL	CERN-OHL-S v2	https://zenodo.org/records/18899461/files/l1_drag_chain_bracket.stl
l1_sensor_vertical_barA	STL	CERN-OHL-S v2	https://zenodo.org/records/18899461/files/l1_sensor_vertical_barA.stl
l1_sensor_vertical_barB	STL	CERN-OHL-S v2	https://zenodo.org/records/18899461/files/l1_sensor_vertical_barB.stl
l1_sensor_housing	STL	CERN-OHL-S v2	https://zenodo.org/records/18899461/files/l1_sensor_housing.stl

Table S4: Layer 2 (pushing and separation) design files

Design filename	File type	Open source license	Location of the file
l2_arm_base	STL	CERN-OHL-S v2	https://zenodo.org/records/18899461/files/l2_arm_base.stl
l2_arm_base_key	STL	CERN-OHL-S v2	https://zenodo.org/records/18899461/files/l2_arm_base_key.stl
arm_end_plate_B_l2	STL	CERN-OHL-S v2	https://zenodo.org/records/18899461/files/arm_end_plate_B_l2.stl
arm_guide_rail_B_l2	STL	CERN-OHL-S v2	https://zenodo.org/records/18899461/files/arm_guide_rail_B_l2.stl
l2_housing	STL	CERN-OHL-S v2	https://zenodo.org/records/18899461/files/l2_housing.stl
l2_latch	STL	CERN-OHL-S v2	https://zenodo.org/records/18899461/files/l2_latch.stl
l2_lever	STL	CERN-OHL-S v2	https://zenodo.org/records/18899461/files/l2_lever.stl
l2_pusher_base	STL	CERN-OHL-S v2	https://zenodo.org/records/18899461/files/l2_pusher_base.stl
l2_pusher	STL	CERN-OHL-S v2	https://zenodo.org/records/18899461/files/l2_pusher.stl
l2_pin	STL	CERN-OHL-S v2	https://zenodo.org/records/18899461/files/l2_pin.stl
l2_spring_postA	STL	CERN-OHL-S v2	https://zenodo.org/records/18899461/files/l2_spring_postA.stl
l2_spring_postB	STL	CERN-OHL-S v2	https://zenodo.org/records/18899461/files/l2_spring_postB.stl

Table S5: Layer 3 (gripping) design files

Design filename	File type	Open source license	Location of the file
l3_gripper	STL	CERN-OHL-S v2	https://zenodo.org/records/18899461/files/l3_gripper.stl
l3_spacer	STL	CERN-OHL-S v2	https://zenodo.org/records/18899461/files/l3_spacer.stl
